# Functional Characterization of *CsBAS1*, *CsSND1*, and *CsIRX6* in Cucumber Defense Against *Meloidogyne incognita*

**DOI:** 10.3390/ijms26052133

**Published:** 2025-02-27

**Authors:** Shihui Li, Xueyun Wang, Lihong Gao, Yongqiang Tian, Si Ma

**Affiliations:** 1 Beijing Key Laboratory of Growth and Development Regulation for Protected Vegetable Crops, College of Horticulture, China Agriculture University, Beijing 100193, China; lshlishihui@163.com (S.L.); wxy15832324269@163.com (X.W.); gaolh@cau.edu.cn (L.G.); 2 State Key Laboratory for Quality and Safety of Agro-Products, Institute of Vegetables, Zhejiang Academy of Agricultural Sciences, Hangzhou 310021, China

**Keywords:** cucumber, vascular bundle, *Meloidogyne incognita*, giant cell

## Abstract

Vascular tissue development plays a pivotal role in plant growth and defense against biotic stress. Root-knot nematodes, particularly *Meloidogyne incognita* (*M. incognita*), are globally distributed phytopathogens that cause severe economic losses in a variety of vascular plants. In this study, three vascular bundle development-related genes, including *CsBAS1*, *CsSND1*, and *CsIRX6*, were identified in cucumber. Tissue-specific expression analysis revealed that *CsSND1* and *CsIRX6* were highly expressed in roots. Infection with *M. incognita* showed dynamic expression changes for *CsBAS1*, *CsSND1*, and *CsIRX6*. Specially, *CsIRX6* and *CsSND1* were upregulated at 14 days post-inoculation (dpi), while *CsBAS1* was downregulated at both 7 dpi and 14 dpi. Tissue localization studies using promoter–GUS constructs demonstrated *pCsBAS1*-*GUS* and *pCsSND1*-*GUS* activity in galls and specific vascular tissues, while *CsIRX6* mRNA was detected in giant cells (GCs) at 14 dpi using *in situ* methods. Virus-induced gene silencing (VIGS) of *CsBAS1*, *CsSND1*, and *CsIRX6* revealed their distinct roles in nematode-induced gall formation. Silencing *CsBAS1* and *CsSND1* resulted in increased root growth and gall size, whereas silencing *CsIRX6* led to reduced gall size. These findings highlight the functional significance of *CsBAS1*, *CsSND1*, and *CsIRX6* in cucumber defense against *M. incognita*, offering insights into the interplay between vascular development and plant defense mechanisms.

## 1. Introduction

Root-knot nematodes (RKNs, *Meloidogyne* spp.) are major phytopathogens with a worldwide distribution, causing substantial economic losses in agricultural production [1]. Annual yield losses due to RKN infestations are estimated to reach billions of dollars [2]. These nematodes predominantly invade plant roots, inducing the formation of root galls that disrupt critical physiological processes, particularly those associated with water and nutrient transport [3]. The vascular system, comprising xylem and phloem, is essential for the transport of water, minerals, and photosynthetic products, thereby supporting normal plant growth and development [4,5]. However, RKN infection leads to significant structural and functional alterations in vascular tissues [6]. Through complex feeding and parasitism mechanisms, RKNs induce cellular re-differentiation and tissue reorganization, resulting in the formation of giant cells (GCs) [7]. These structural changes compromise vascular functionality, severely impairing the transport of water and nutrients [8], which manifests as wilting, stunted growth, and reduced crop productivity in infected plants [9]. Understanding the mechanisms underlying vascular system disruption during RKN infection is crucial for advancing our knowledge of plant–nematode interactions. Moreover, such insights are essential for the development of targeted resistance strategies to mitigate the impact of RKNs on agricultural sustainability and global food security. Despite recent progress in elucidating the structure and function of the vascular system, the specific molecular pathways and genes that play important roles in vascular development in the context of pathogen-induced stress remain poorly understood.

The development and maintenance of vascular tissues are orchestrated by intricate genetic and hormonal networks [9]. Brassinosteroids (BRs), a class of steroid hormones, play a critical role in regulating vascular differentiation, as well as plant growth and stress responses [10,11]. BR-regulated genes, such as *DWF4* and *CYP85A3*, are directly involved in vascular tissue differentiation, particularly in xylem and phloem formation [12,13]. Overexpression of *PtoDWF4* or *PtCYP85A3* in poplars enhances xylem growth, resulting in an increase in biomass yield [12,13]. In watercress (*Nasturtium officinale*), the application of the BR-specific biosynthesis inhibitor Brassinazole (Brz) significantly suppresses secondary xylem development while enhancing phloem cell development [14]. The BAS1 gene, encoding the P450 cytochrome oxidase (CYP72B1), acts as a negative regulator of BR metabolism [15]. Overexpression of *AtBAS1* in Arabidopsis reduces BR levels, resulting in impaired xylem and phloem development [16]. Homologous genes involved in BR regulation have been identified in other species, such as *OsCYP734A* in rice (*Oryza sativa*) and *LeCYP734A7* in tomato (*Solanum lycopersicum*) [17,18,19]. These genes function similarly to *AtBAS1*, further highlighting the conserved role of BR-related pathways in vascular development across different plant species [17,18,19].

In addition to hormonal regulation, transcription factors (TFs) play a crucial role in orchestrating vascular development [20]. Members of the NAC (NAM, ATAF1/2, CUC2) family of transcription factors have emerged as key regulators of secondary cell wall biosynthesis and xylem differentiation [21,22]. For instance, SND1 (Secondary Wall-Associated NAC Domain Protein 1) and its close functional relatives VND6 and VND7 are essential for the differentiation and programmed cell death of xylem vessel cells, processes critical for vascular functionality [23,24,25,26]. In Arabidopsis, *AtSND1* is specifically expressed in the interfascicular regions and xylary fibers of the stem, where it regulates fiber thickening [25,27,28]. In rice, mutations in *VND6* result in reduced cellulose content, thinning of secondary walls, and impaired water transport in the xylem [29]. Furthermore, SND1, in collaboration with VND6 and VND7, influences the expression of *MYBs*, thereby upregulating genes involved in the biosynthesis of secondary wall components, including cellulose, lignin, and xylan [24]. Lignin, xylan, and cellulose are vital components of the secondary cell wall and contribute significantly to plant defense mechanisms, particularly within vascular tissues [30]. These components not only reinforce structural integrity but also enhance resistance to environmental stresses, underscoring the importance of NAC TFs in vascular development and plant adaptation.

The *COBRA* gene family, which encodes glycosylphosphatidylinositol (GPI)-anchored proteins, is another critical player in vascular development [31]. COBRA-like proteins are involved in cellulose and hemicellulose deposition in the secondary cell wall, processes vital for vascular tissue integrity [32]. *IRX6* encodes a member of the COBRA-like family, which is integral to secondary cell wall biosynthesis, particularly in xylem tissues [33]. In Arabidopsis, loss-of-function mutants of *AtIRX6* result in vessel collapse as well as reduced cellulose levels and cell wall sugar content [33]. In rice, mutations in *COBL* lead to significantly thinner cell walls in sclerenchyma and vascular bundle cells, accompanied by a decrease in cellulose content [34]. Similarly, the *bk2* mutant in maize exhibits a drastic reduction in cell wall thickness in the stem [35]. In tomatoes, the *SlCOBRA*-like gene is crucial for the structural integrity of fruit epidermal cell walls, contributing to enhanced fruit firmness and extended shelf life [36]. Additionally, COBRA genes have been implicated in stress resistance [37,38]. In rice and sweet sorghum, COBRA family members are associated with improved resistance to drought and salt stress [37,38].

Although homologs of *CsBAS1*, *CsSND1*, and *CsIRX6* have been well characterized in model plants like Arabidopsis for their roles in vascular development and secondary cell wall biosynthesis, their functions in crop species, particularly under biotic stress conditions, remain poorly understood. This study characterized the roles of *CsBAS1*, *CsSND1*, and *CsIRX6* in response to nematode stress in cucumber, providing new insights into the interplay between vascular development and plant defense mechanisms.

## 2. Results

### 2.1. Identification and Phylogenetic Analysis of Cucumber Vascular Bundle Development-Related Genes

By querying the cucumber genome database, six candidate genes associated with vascular bundle development were identified: *Csa2G006030* (*CsBAS1*), *Csa5G148470* (*CsSND1*), *Csa2G070320* (*CsIRX3*), *Csa6G088080* (*CsIRX5*), *Csa1G015700* (*CsIRX6*), and *Csa3G081360* (*CsIRX8*). Phylogenetic trees for the BAS, SND, and IRX gene families were constructed using sequences from Arabidopsis, cucumber, and tomato. This analysis revealed that *CsBAS1*, *CsSND1*, *CsIRX3*, *CsIRX5*, *CsIRX6*, and *CsIRX8* are most closely related to *AtBAS1* (*AT2G26710*), *AtSND1* (*AT1G32770*), *AtIRX3* (*AT5G17420*), *AtIRX5* (*AT5G44030*), *AtIRX6* (*AT5G15630*), and *AtIRX8* (*AT5G54690*) in Arabidopsis, respectively (Supplemental Figure S1).

### 2.2. Tissue-Specific Expression Analysis of CsBAS1, CsSND1, and CsIRXs

**Figure 1:**
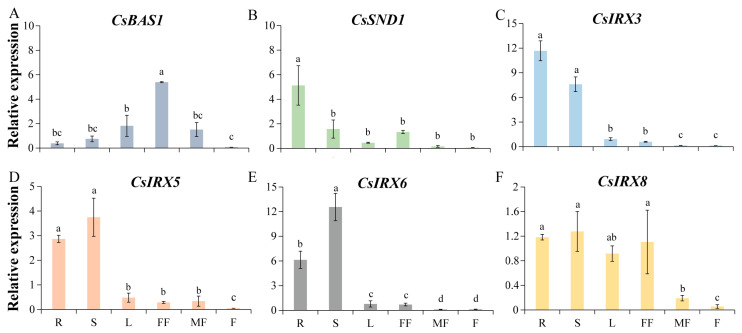
Expression profiles of vascular bundle development-related genes in various cucumber tissues. (**A**) *CsBAS1*, (**B**) *CsSND1*, (**C**) *CsIRX3*, (**D**) *CsIRX5*, (**E**) *CsIRX6*, and (**F**) *CsIRX8*. Significant differences (*p* < 0.05) are indicated by different letters above the bars (Duncan test). Data are presented as mean ± SE (n = 3). R, root; S, stem; L, leaf; FF, female flower; MF, male flower; F, fruit.

To explore the tissue-specific expression patterns of *CsIRXs*, *CsBAS1*, and *CsSND1*, RNA was extracted from cucumber roots, stems, leaves, female flowers, male flowers, and fruits. As shown in Figure 1, *CsIRX3* and *CsSND1* exhibited the highest expression levels in roots. *CsIRX5*, *CsIRX6*, and *CsIRX8* were predominantly expressed in roots and stems. *CsBAS1* showed the highest expression in female flowers.

*M. incognita* induces highly specific feeding sites in plant roots. Second-stage juveniles (J2s) of *M. incognita* mechanically destroy and invade the roots at the elongation zone just behind the root tip. Upon infection, J2s swim intercellularly towards the vascular cylinder to select suitable cells for the formation of feeding sites [39]. To investigate the effects of *M. incognita* infection on vascular tissue development, root gall samples were collected at 7 to 42 days post-inoculation (dpi), paraffin-embedded, and sectioned for analysis. At 7 dpi, transverse sections of galls showed the presence of J2s that had established feeding sites and formed GCs, though no notable vascular tissue differentiation was observed (Supplemental Figure S2A). By 14 and 21 dpi, the number and size of GCs increased notably (Supplemental Figure S2B,C). By 28-35 dpi, the outer layers of the root thinned, and new vascular tissue began to form. This was accompanied by the expansion and proliferation of GCs and surrounding tissues, indicating signs of vascularization (Supplemental Figure S2D,E). At 42 dpi, some GCs exhibited signs of nutrient depletion, leading to vacuolation formation and disorganized vascular tissue (Supplemental Figure S2F).

**Figure 2:**
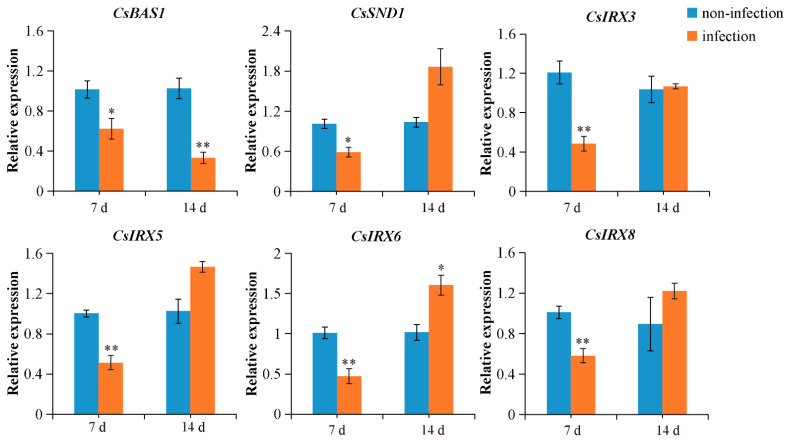
Expression levels of vascular bundle development-related genes in cucumber roots, with or without *M. incognita* infection, were analyzed at 7 dpi and 14 dpi. Significant differences are indicated as follows: * *p* < 0.05, ** *p* < 0.01 (Student’s *t*-test). Data are shown as mean ± SE (n = 3).

To assess the responsiveness of vascular tissue development-related genes to *M. incognita* infection in cucumber, the expression levels of *CsIRX3*, *CsIRX5*, *CsIRX6*, *CsIRX8*, *CsBAS1*, and *CsSND1* in galls were analyzed using qRT-PCR, with uninfected tissues serving as the control. Based on the developmental stages of the nematodes, we selected 7 dpi and 14 dpi for our study, which are key time points for both the early establishment and later maturation of the nematode-induced galls [40]. As shown in Figure 2, *CsIRX3*, *CsIRX5*, *CsIRX6*, and *CsIRX8* expression levels were downregulated at 7 dpi, with only *CsIRX6* showing upregulation at 14 dpi, which led to its selection as the primary target for further investigation. *CsBAS1* expression was significantly downregulated at both 7 and 14 dpi. Similarly, *CsSND1* showed a significant decrease in expression at 7 dpi.

### 2.3. Tissue Localization of CsBAS1, CsSND1, and CsIRX6 in Cucumber Post-Infection by M. incognita

**Figure 3:**
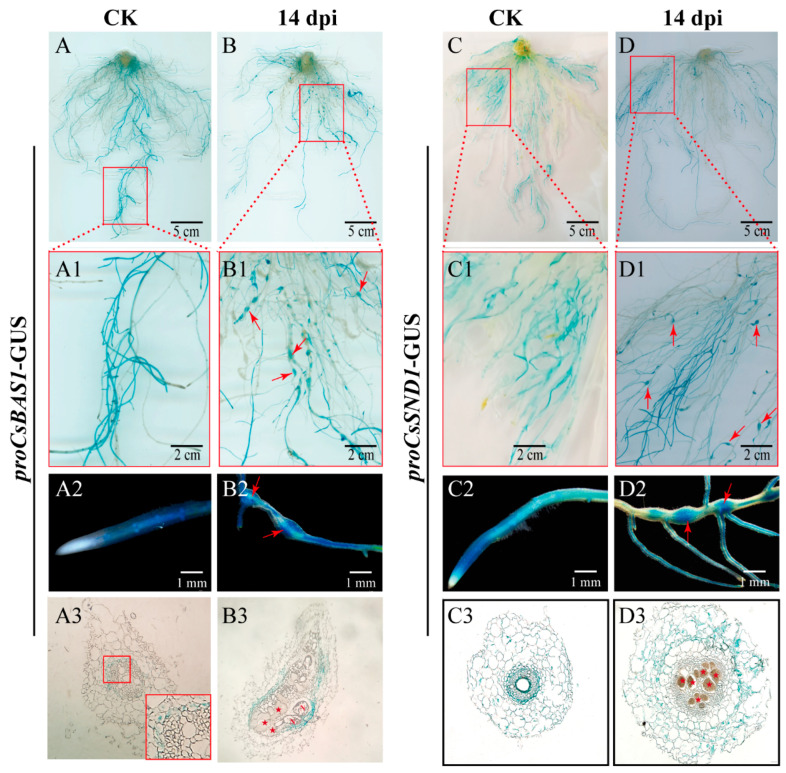
Tissue localization of *CsBAS1* and *CsSND1* in cucumber roots, with or without *M. incognita* infection, was detected at 14 dpi. (**A**–**D**) GUS staining patterns in hairy roots harboring *proCsBAS1::*GUS and *proCsSND1::*GUS constructs, with (**A**,**C**) or without (**B**,**D**) *M. incognita* infection. Scale bar = 5 cm. (**A1**–**D1**) Galls (indicated by red arrows) are observed in hairy roots expressing *proCsBAS1::*GUS (**B1**) and *proCsSND1::*GUS (**D1**) following nematode infection. (**A2**–**D2**) Blue GUS signals resulting from β-glucuronidase activity are present in roots (**A2**,**C2**) without *M. incognita* infection and galls (**B2**,**D2**) induced by *M. incognita*, highlighting tissue localization. (**A3**–**D3**) GUS signals in 10 μm paraffin-embedded sections of roots without (**A3**,**C3**) or with (**B3**,**D3**) *M. incognita* infection for *proCsBAS1::*GUS and *proCsSND1::*GUS. Annotations: N = nematode; red arrows indicate galls; pentagrams indicate giant cells. The red frame represents an enlarged view of A3.

**Figure 4:**
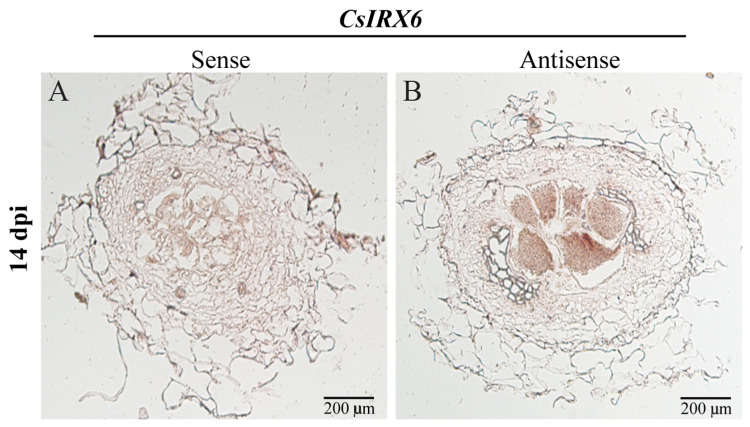
*In situ* hybridization analysis of *CsIRX6* mRNA in cucumber roots with *M. incognita* infection at 14 dpi. Cross-sections of galls are hybridized with digoxigenin-labeled *CsIRX6* sense (**A**) or antisense (**B**) RNA probes. The hybridization signals are shown as red-brown. Scale bar = 200 μm. Pentagrams indicate giant cells.

Based on the observed changes in expression levels under *M. incognita* infection, *CsBAS1*, *CsSND1*, and *CsIRX6* were selected for further investigation. To confirm their expression in cucumber roots infected by *M. incognita*, hairy root transformation assays were performed using *Agrobacterium rhizogenes* carrying promoter–GUS fusion constructs. Strong GUS staining driven by *proCsBAS1::*GUS was observed in cucumber roots and galls at 14 d, but not in root tips (Figure 3(A2,B2)). Sectioning revealed that *CsBAS1* expression was localized in the phloem tissue and surrounding areas of GCs (Figure 3(A3,B3)). The *proCsSND1::*GUS construct displayed a similar staining pattern, with signals present in the cortex but absent from GCs within galls (Figure 3(C3,D3)). Due to the absence of *proCsIRX6::*GUS transgenic hairy roots, GUS staining assays were not conducted. Instead, *in situ* hybridization experiments were performed, revealing that *CsIRX6* mRNA accumulated within GCs at 14 dpi (Figure 4).

### 2.4. Functional Analysis of CsBAS1, CsSND1, and CsIRX6 in Cucumber Infected by M. incognita

A *TRSV*-mediated virus-induced gene silencing (VIGS) system, previously used to study the role of genes in cucumber [41,42], was employed to analyze vascular tissue development-related genes. The phytoene desaturase (PDS) gene was used as a positive control (Supplemental Figure S3A), and the silencing efficiency of *CsBAS1*, *CsSND1*, and *CsIRX6* was evaluated in each treated root (Supplemental Figure S3B).

**Figure 5:**
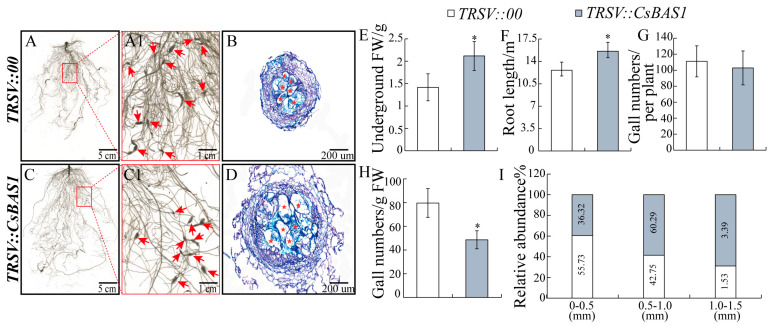
Suppression of *CsBAS1* through VIGS promoted root development and enhanced gall size at 14 dpi with nematode infection. (**A**,**A1**) Root phenotype of control plants (TRSV*::00*) inoculated with *M. incognita*. (**C**,**C1**) Root phenotype of *CsBAS1*–silenced plants (TRSV*::CsBAS1*) inoculated with *M. incognita*. (**B**,**D**) Gall sections stained with toluidine blue. Pentagram indicates giant cell. Comparison of underground weight (**E**), root length (**F**), gall numbers per plant (**G**), gall numbers/g FW (**H**), and gall size abundance (**I**) in TRSV*::00* and TRSV*::CsBAS1* inoculated with *M. incognita* at 14 dpi. Significant differences are indicated as follows: * *p* < 0.05 (Student’s *t*–test). Data are shown as mean ± SE (n = 15).

To investigate the role of *CsBAS1*, the *TRSV::CsBAS1* bacteria were used to infect cucumber cotyledons, generating *CsBAS1*-silenced lines. Among the silenced plants, 38.46% exhibited a silencing efficiency above 70%, 30.77% between 40% and 70%, and 23.08% below 40% (Supplemental Figure S3B). Plants with over 40% silencing efficiency were analyzed further. *TRSV::CsBAS1* plants exhibited significantly increased root fresh weight and root length compared to *TRSV::00* plants (Figure 5E,F). Although the number of galls per plant was similar between the two groups, the gall number per gram of root was lower in *TRSV::CsBAS1* plants (Figure 5G,H). Gall size analysis revealed that 60.29% of galls in *TRSV::CsBAS1* plants had diameters of 0.5–1.0 mm, compared to 42.75% in the controls (Figure 5I). Conversely, 36.32% of galls in *TRSV::CsBAS1* plants had diameters of 0–0.5 mm, compared to 55.73% in controls (Figure 5I). Subsequently, paraffin sections were prepared from cucumber roots infected with *M. incognita* at 14 dpi. Histological sections showed larger GCs in *TRSV::CsBAS1* plants compared to controls (Figure 5B,D). These results indicate that silencing *CsBAS1* promotes GCs development.

**Figure 6:**
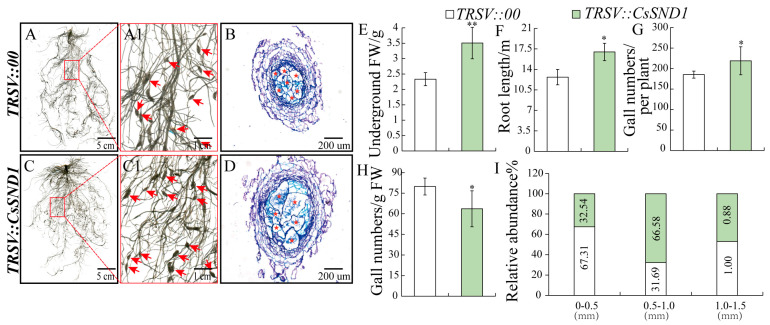
Suppression of *CsSND1* through VIGS promoted root development and enhanced gall size at 14 dpi with nematode infection. (**A**,**A1**) Root phenotype of control plants (*TRSV::00*) inoculated with *M. incognita*. (**C**,**C1**) Root phenotype of *CsSND1*–silenced plants (*TRSV::CsSND1*) inoculated with *M. incognita*. (**B**,**D**) Gall sections stained with toluidine blue. Pentagram indicates giant cell. Comparison of underground weight (**E**), root length (**F**), gall numbers per plant (**G**), gall numbers/g FW (**H**), and gall size abundance (**I**) in TRSV*::00* and TRSV*::CsSND1* inoculated with *M. incognita* at 14 dpi. Significant differences are indicated as follows: * *p* < 0.05, ** *p* < 0.01 (Student’s *t*-test). Data are shown as mean ± SE (n = 15).

To explore the role of *CsSND1* in response to *M. incognita* in cucumber, *TRSV::CsSND1*-silenced lines were generated using a VIGS system. Among the obtained silenced lines, 29.41% exhibited a silencing efficiency above 70%, and 41.18% between 40% and 70% (Supplemental Figure S3C). Plants with over 40% silencing efficiency were selected for analysis. Compared to *TRSV::00* control plants, *TRSV::CsSND1* plants showed a significant increase in root length and root fresh weight (Figure 6E,F). The number of galls was significantly higher in *TRSV::CsSND1* plants than in the controls (Figure 6G). Regarding the distribution of gall sizes, 66.58% of galls in *TRSV::CsSND1* plants had diameters of 0.5–1.0 mm, compared to 31.69% in controls (Figure 6I). Histological sections of galls from both control and *TRSV::CsSND1* plants revealed that transient silencing of *CsSND1* increased gall diameter (Figure 6B,D). These results suggest that transient silencing of *CsSND1* enhances gall development and promotes *M. incognita* infection.

**Figure 7:**
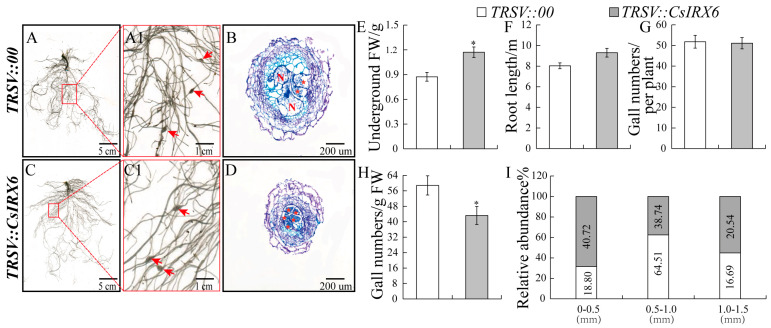
Suppression of *CsIRX6* through VIGS promoted root development and inhibited gall size at 14 dpi with nematode infection. (**A**,**A1**) Root phenotype of control plants (*TRSV::00*) inoculated with *M. incognita*. (**C**,**C1**) Root phenotype of *CsIRX6*–silenced plants (*TRSV::CsIRX6*) inoculated with *M. incognita*. (**B**,**D**) Gall sections stained with toluidine blue. Pentagram indicates giant cell. Comparison of underground weight (**E**), root length (**F**), gall numbers per plant (**G**), gall numbers/g FW (**H**), and gall size abundance (**I**) in *TRSV::00* and *TRSV::CsIRX6* inoculated with *M. incognita* at 14 dpi. Significant differences are indicated as follows: * *p* < 0.05 (Student’s *t*–test). Data are shown as mean ± SE (n = 15).

Similarity, to investigate the function of *CsIRX6* in cucumber root growth following *M. incognita* infection, *TRSV::CsIRX6*-silenced plants were generated using the VIGS system. Among the silenced lines, 24.53% showed a silencing efficiency above 70%, 52.83% between 40% and 70%, and 22.64% below 40% (Supplemental Figure S3D). Plants with over 40% silencing efficiency were analyzed further. Compared to controls, *TRSV::CsIRX6* plants exhibited significant increases in root fresh weight, with no significant differences in root length or gall number (Figure 7E–G). Gall size distribution showed that *TRSV::CsIRX6* plants had more galls in the 0–0.5 mm range (40.72%) compared to controls (18.8%) (Figure 7I). Histological analysis revealed reduced GC sizes in *TRSV::CsIRX6* plants, resulting in smaller galls (Figure 7B,D,I). These results indicate that transient silencing of *CsIRX6* inhibits *M. incognita* infection by suppressing GC development while promoting root growth in cucumber.

## 3. Discussion

RKNs bring a significant threat to agriculture, causing substantial economic losses and yield reductions annually [43,44,45]. During RKN infection, cells near the infection site undergo expansion and reorganization, leading to disorganization of the vascular structure, such as vessel blockage and decreased conductive function, which in turn affects the transport of nutrients [46]. Additionally, xylem and phloem cells in galls may expand, and localized vascular necrosis may occur [46,47]. Vascular tissue plays a critical role as a site for pathogen nutrient acquisition following host invasion [3]. Therefore, studying vascular tissue development, *de novo* vascularization of the phloem and xylem, and defense mechanisms against nematodes is essential. However, research on the detailed interactions between nematode infection and genes related to vascular tissue development remains limited. Here, we studied the gene expression, tissue localization, and potential roles of *CsBAS1*, *CsSND1*, and *CsIRX6* in vascular tissue development during *M. incognita* infection.

The anatomic characteristics of root galls in cucumber revealed a typical process of nematode feeding site formation (Supplemental Figure S2), consistent with previous studies [3,8,28,48]. At 14 dpi, 5–7 GCs were observed as feeding sites for female nematodes (Supplemental Figure S2B). GCs serve as the sole nutrient source for nematode development and reproduction [46]. Disruption of feeding sites in other crops has been shown to confer resistance to nematodes [49,50,51]. Nematode infection interferes with vascular continuity, leading to the *de novo* formation of phloem and xylem components [3,52,53]. As shown in Supplemental Figure S2E,F, cells near the feeding sites displayed deformation, while surrounding tissues underwent vascularization, leading to disorganization of the vascular cylinder. These results indicate that nematode infection disrupts normal vascular development, induces new vascular tissue formation, and may impair nutrient transport.

The vascular tissue development-related genes *CsBAS1*, *CsSND1*, and *CsIRX6* were selected due to their higher responsiveness to nematode infection at 7 dpi and 14 dpi (Figure 2). Phylogenetic analysis (Supplemental Figure S1) suggests that the functions of *BAS1*, *SND1*, and *IRX6* are conserved between cucumber and Arabidopsis, where they may contribute to vascular tissue development [25,33,54,55]. Furthermore, expression analysis revealed that *CsCsBAS1*, *CsSND1*, and *CsIRX6* were significantly reduced in galls following nematode infection at 7 dpi (Figure 2). GUS staining in galls was consistent with previous research findings [51,56]. Cross-sections of galls at 14 dpi found that *CsBAS1* was expressed in the phloem tissue near GCs, while *CsSND1* was primarily detected in the cortex (Figure 3). *In situ* hybridization signals of *CsIRX6* mRNA accumulated predominantly in the GCs (Figure 4). These results indicate the potential roles of *CsBAS1*, *CsSND1*, and *CsIRX6* in vascular development and nematode infection.

BAS1 is a BR-inactivating enzyme that regulates BR metabolism [57,58]. Overexpression of *BAS1* in Arabidopsis and tobacco decreases brassinolide content while increasing the accumulation of 26-hydroxybrassinolide [57]. Disruption of the balance of BR hormone levels affects root length, cell elongation, division, and differentiation [59,60]. In this study, we found that in *CsBAS1*-silenced lines, root length and fresh weight were significantly increased, and gall size was larger than that of the control at 14 dpi (Figure 5). Furthermore, the number of galls per gram of root was greatly decreased in *CsBAS1*-silenced lines, although the number of galls showed no significant difference (Figure 5G,H). Based on these results, we speculate that silencing *CsBAS1* increases cucumber susceptibility to *M. incognita*, possibly by modulating BR levels.

Previous studies have shown that *SND1* plays an important role in the formation and development of vascular bundles and secondary cell walls by regulating cellulose and lignin biosynthesis [61,62,63]. In cotton stems, silencing both *SND1* and *NST1* results in developmental defects in the xylem and phloem [62]. Overexpression of *CpSND1* in Arabidopsis leads to inhibited plant growth, a significant increase in secondary wall thickness, enhanced lignin content, and the upregulation of genes involved in cellulose and lignin biosynthesis [64]. In our study, we observed that silencing *CsSND1* resulted in larger and more galls (Figure 6). This phenomenon was closely related to the reduced function of *CsSND1*, which affected lignin content and cell wall synthesis, disrupted the physical barrier, and ultimately induced gall formation [65]. Studies have shown that structural modifications of the cell wall can alter patterns of cell division and expansion [66]. We hypothesize that silencing *CsSND1* may alter secondary wall development, thereby affecting cell division and expansion in the root apical meristem and promoting root elongation (Figure 6E). Our results suggest that *CsSND1* plays a positive role in gall development by remodeling the structure of the cell wall.

*IRX6*/*COBL4* plays a key role in the deposition of cellulose in the cell wall [61,67]. In this study, we found that transient silencing of *CsIRX6* produced smaller galls (Figure 7B,D,I). Consistent with previous studies, *atcobl4* mutants exhibited collapsed xylem vessels, which disrupted the development of vascular bundles [33]. This disruption may significantly limit the ability of nematodes to acquire nutrients from the host. *COBL4* is localized to the secondary cell wall bands of protoxylem cells and co-expresses with genes involved in secondary cell wall biosynthesis [33,67]. Our results showed that *CsIRX6* was strongly expressed in GCs following nematode infection (Figure 4). GCs are typically derived from transformed host vascular cells that gradually proliferate and expand, undergoing significant changes in their cell walls and structure [46]. *CsIRX6* may influence the structure of GCs by regulating the content of cellulose and xylan. Thus, *CsIRX6* likely regulates cucumber susceptibility to *M. incognita* by modulating GC development.

In this study, we investigated the relationship between vascular development and root-knot nematode infection in plants. Specifically, we focused on genes implicated in vascular development. By combining phylogenetic analysis, tissue localization, nematode infection assays, and gene silencing, we identified the roles of CsBAS1, CsSND1, and CsIRX6 in cucumber infected with nematodes. However, the potential interactions and underlying mechanisms between these three genes remain unclear and will be addressed in future studies.

## 4. Materials and Methods

### 4.1. Plant Materials and Nematode Propagation

‘*Xintaimici*’, a homozygous inbred line of cucumber (*Cucumis sativus* L.) with a stable genetic background that is widely used in genetic transformation and functional studies, with high susceptibility to *M. incognita*, was studied. Seeds were sown in pots containing a sand–vermiculite mixture (1:1 *v*/*v*). The plants were grown under controlled conditions in a growth chamber with a 16 h light/8 h dark photoperiod, maintaining a temperature regime of 25 °C during the day and 18 °C at night. Cucumber samples from the root, stem, leaf, female flower, male flower, and fruit were collected for tissue-specific expression assays.

*M. incognita* were propagated on the roots of water spinach (*Ipomoea aquatica* Forsk cv. Liuye), grown in soil. Nematode eggs were extracted from the root galls and incubated in water at 28 °C in the dark for approximately 7 days, allowing them to hatch. Cucumber seedlings, approximately four weeks old, were inoculated with 200 freshly hatched pre-J2s, and controls were treated with water. Root galls were collected at 7, 14, 21, 28, 35, and 42 dpi. Whole root samples from nematode-infested and non-infested cucumbers were collected at 7 and 14 dpi.

### 4.2. Phylogenetic Analysis

To further investigate the evolutionary relationships of BASs, SNDs, and IRXs in cucumber and other species, protein sequences of BASs, SNDs, and IRXs from *Arabidopsis thaliana*, *Cucumis sativus*, and *Solanum lycopersicum* were retrieved from the Arabidopsis database (https://www.arabidopsis.org/ accessed on 12 February 2025), the cucumber Chinese Long v2 genome database (http://cucurbitgenomics.org/organism/2 accessed on 12 February 2025), and the tomato database (https://solgenomics.net/about/tomato_project_overview.pl accessed on 12 February 2025), respectively. Phylogenetic analysis was performed using MEGA 5.0 software, applying the neighbor-joining method with a bootstrap analysis of 1000 replicates.

### 4.3. Agrobacterium Rhizogenes Mediated Transgenic Hairy Roots and GUS Analysis

Promoter fragments of *CsBAS1* (*Csa2G006030*, 2535 bp) and *CsSND1* (*Csa5G148470*, 1834 bp) were cloned into the pCAMBIA1391 vector using primers listed in Supplemental Table S1. The recombinant plasmids were introduced into *Agrobacterium rhizogenes* strain K599 to generate transgenic hairy roots, following the method described by Zhang et al. [68]. Briefly, *Agrobacterium* cultures were plated on selective medium (rifampicin 50 µg/mL, kanamycin 50 µg/mL) and incubated at 28 °C for 3 days. The *Agrobacterium* suspension was adjusted to an OD_600_ of 0.6–0.8 and injected beneath the cotyledons of cucumber seedlings. Hairy roots grew within approximately three weeks, after which seedlings were transplanted into pots with a 1:1 mixture of sand and vermiculite. Half of the seedlings were inoculated with 200 freshly hatched pre-J2s, and the others served as controls. Root samples were collected at 7 and 14 dpi.

GUS staining was performed according to the kit instructions (Coolaber, Beijing, China, CAT#: SL7160). Briefly, all hairy roots were immersed in GUS staining solution at 37 °C for 3 h, followed by decolorization in 70% ethanol. The stained roots were then observed under a stereomicroscope.

### 4.4. Histological Analysis of Root Galls

Paraffin sections of the root galls were prepared as described by Zhang et al. [68], with slight modifications. Briefly, collected root galls were fixed in 50% FAA solution and subjected to vacuum infiltration for 20 min, which was repeated twice. Samples were stained with eosin and toluidine blue, then observed under an Olympus B73 microscope.

### 4.5. In Situ Hybridization Assay

Since the promoter of *CsIRX6* (*Csa1G015700*) could not be successfully cloned, an alternative approach, *in situ* hybridization, was employed to evaluate the mRNA expression of the *CsIRX6* in root galls. The experiment was conducted as described by Sui et al. [69], with modifications. Fresh cucumber root galls were prepared and fixed with 50% FAA and subjected to vacuum infiltration for 15–30 min until the samples settled at the bottom. Then, the medium was replaced with fresh fixative medium and continuous shaking was applied at 4 °C overnight. Afterward, we proceeded with embedding, sectioning, and mounting the sections, followed by the hybridization experiment. Digoxigenin-labeled sense and antisense RNA probes were generated by PCR amplification using SP6 and T7 RNA polymerase (Basel, Switzerland, Roche, 10881767001). The primers are listed in Supplemental Table S1.

### 4.6. RNA Extraction and Quantitative Real-Time PCR

Total RNA was extracted using the Eastep Super Isolation Kit (Beijing, China, CAT#: LS1040). Subsequently, 1 µg of total RNA was used for cDNA synthesis with the HiScript II QRT SuperMix kit with gDNA Wiper for qPCR (Vazyme, Beijing, China, CAT#: R423-01).

qRT-PCR was performed with SYBR^®^ Green I ChamQ SYBR qPCR Master Mix (Vazyme, Beijing, China, CAT#: Q712) on an ABI 7500 Real-Time PCR System (Applied Biosystems, Waltham, MA, USA). *CsUBI* (*Csa2G036600*) and *CsTublin* (*Csa4G000580*) were used as the reference genes for the nematode infestation experiment and tissue expression assay, respectively [70,71]. The relative expression levels of genes were calculated using the 2^−ΔΔCT^ method [72]. Primers are listed in Supplemental Table S1.

### 4.7. TRSV-Mediated VIGS Transient Transformation System

Specific fragments from the CDS regions of *CsBAS1* (650 bp), *CsSND1* (300 bp), and *CsIRX6* (184 bp) were cloned into the pTRSV2 vector and introduced into *Agrobacterium tumefaciens* strain GV3101. Virus-induced gene silencing (VIGS) in cucumber was performed as described by Fang et al. [42]. Briefly, cucumber seeds were surface-sterilized and germinated on MS medium for 2 days. *Agrobacterium* cultures carrying pTRSV1 and pTRSV2 (containing the targeted gene fragments) were mixed, and cucumber cotyledons were inoculated and then cultured in the dark for 4–5 days. The seedlings were then transplanted into pots containing a 1:1 mixture of sand and vermiculite. For controls, a combination of empty pTRSV1 and pTRSV2*::CsPDS* was used as the positive control, while pTRSV1 with pTRSV2*::00* was used as the negative control. Approximately 200 freshly hatched pre-J2s were inoculated into cucumber plants expressing TRSV2*::CsBAS1*, TRSV2*::CsSND1*, TRSV2*::CsIRX6*, or the control TRSV2*::00* once the leaves of plants expressing TRSV2*::CsPDS* began to show whitening. At 14 dpi, root samples were collected, RNA-extracted, and reverse-transcribed for qRT-PCR. Silencing efficiency (%) = ($1-\frac{Expression\;level\;in\;TRSV1}{Expression\;level\;in\;TRSV2::target\;gene}$) × 100. Primer sequences are listed in Supplemental Table S1. Root morphological traits, including root fresh weight and root length, were analyzed using the Epson Perfection V850 Pro software (Epson Co., Ltd, Beijing, China). The gall numbers per plant, gall numbers per gram (FW) of root, and the proportion of galls of different sizes (0–0.5 mm, 0.5–1 mm, 1.0–1.5 mm) were further measured.

### 4.8. Statistical Analysis

All experiments were conducted with a minimum of three independent biological replicates. Experimental data are presented as mean ± standard error (SE). Statistical significance was determined using Student’s *t*-test with significance levels indicated as * *p* < 0.05 and ** *p* < 0.01.

## 5. Conclusions

In conclusion, this study explored the functions of vascular tissue development-related genes *CsBAS1*, *CsSND1*, and *CsIRX6* during *M. incognita* infection in cucumber. These genes exhibit significant induction and are specially expressed in galls during nematode infection, supporting their involvement in host–pathogen interaction (Figure 3 and Figure 4). Gene silencing of *CsBAS1*, *CsSND1*, and *CsIRX6* demonstrated their individual contributions to nematode-induced gall formation (Figure 5, Figure 6 and Figure 7).

Based on our results, we suggest that targeting genes involved in vascular bundle development, such as *CsBAS1*, *CsSND1*, and *CsIRX6*, could be a potential strategy for improving nematode resistance. Additionally, manipulating these genes through genetic engineering or breeding programs may enhance the structural integrity of plant tissues and limit nematode feeding site formation, leading to more resilient crops. Moreover, further research on the interactions between these genes and other defense pathways could offer new avenues for developing crops with improved resistance to nematode infestations.

## Data Availability

All data are presented in the main manuscript and the additional Supplemental Files.

## Supplementary Materials

The supporting information can be downloaded at https://www.mdpi.com/article/10.3390/ijms26052133/s1.
