# Supplementary material for: Functional Characterization of CsBAS1, CsSND1, and CsIRX6 in Cucumber Defense Against Meloidogyne incognita
Source: Int J Mol Sci. 2025 Feb 27;26(5):2133. doi: 10.3390/ijms26052133 (PMC11900111; doi:10.3390/ijms26052133)
Supplement: Supplementary file 1 [file ijms-26-02133-s001.zip › Supplemental figures-2025.1.12@.pdf]

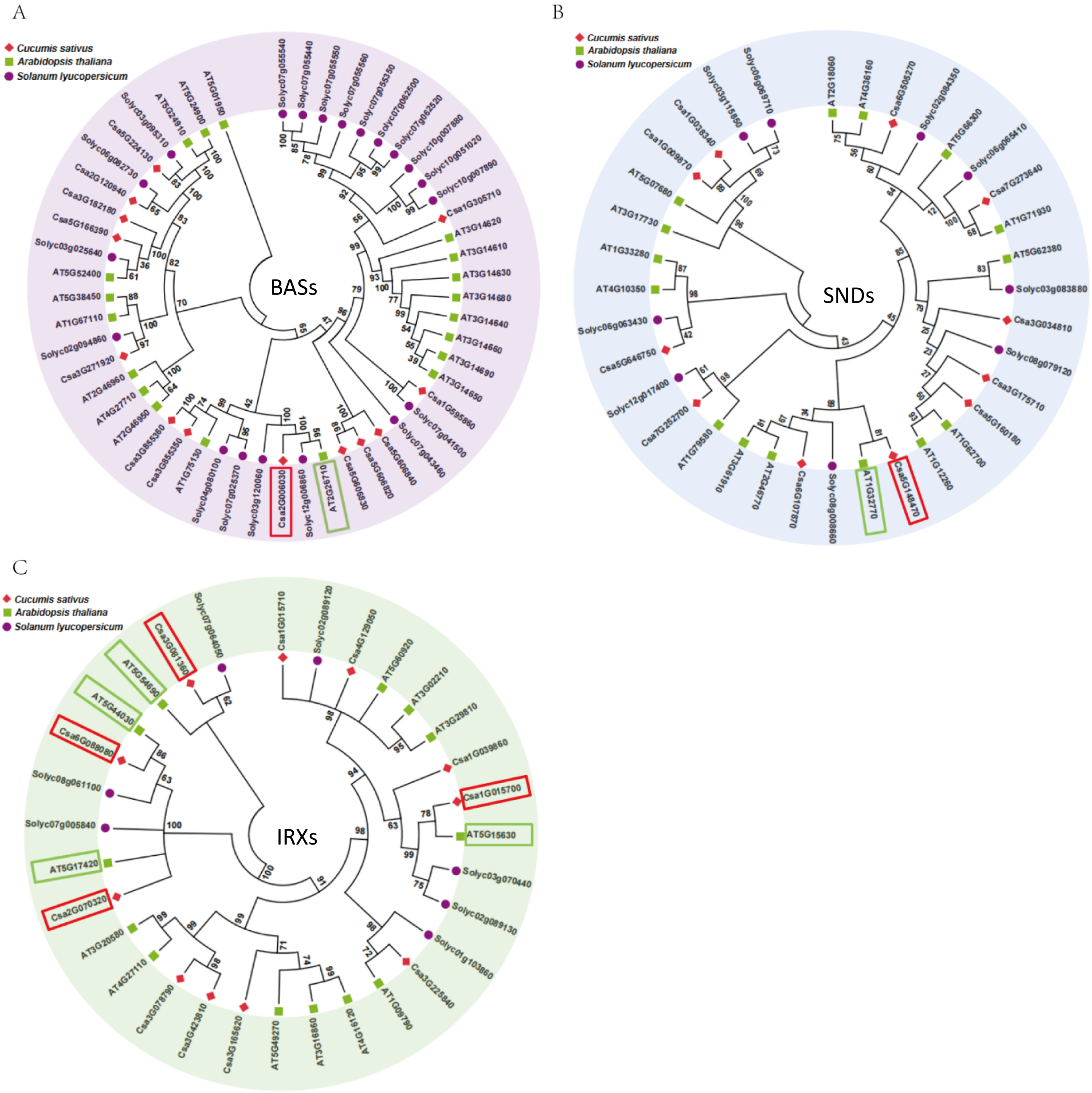

Supplemental figure S1 Phylogenetic analysis of CsBASs (A), CsSNDs (B), and CsIRXs (C) from cucumber (Cs), tomato (Sl), and Arabidopsis (At). A phylogenetic tree is constructed using neighbor-joining method with 1000 bootstrap replicates. The red box represents the gene ID of the cucumber under investigation, while the green box represents the homologous gene ID of Arabidopsis.

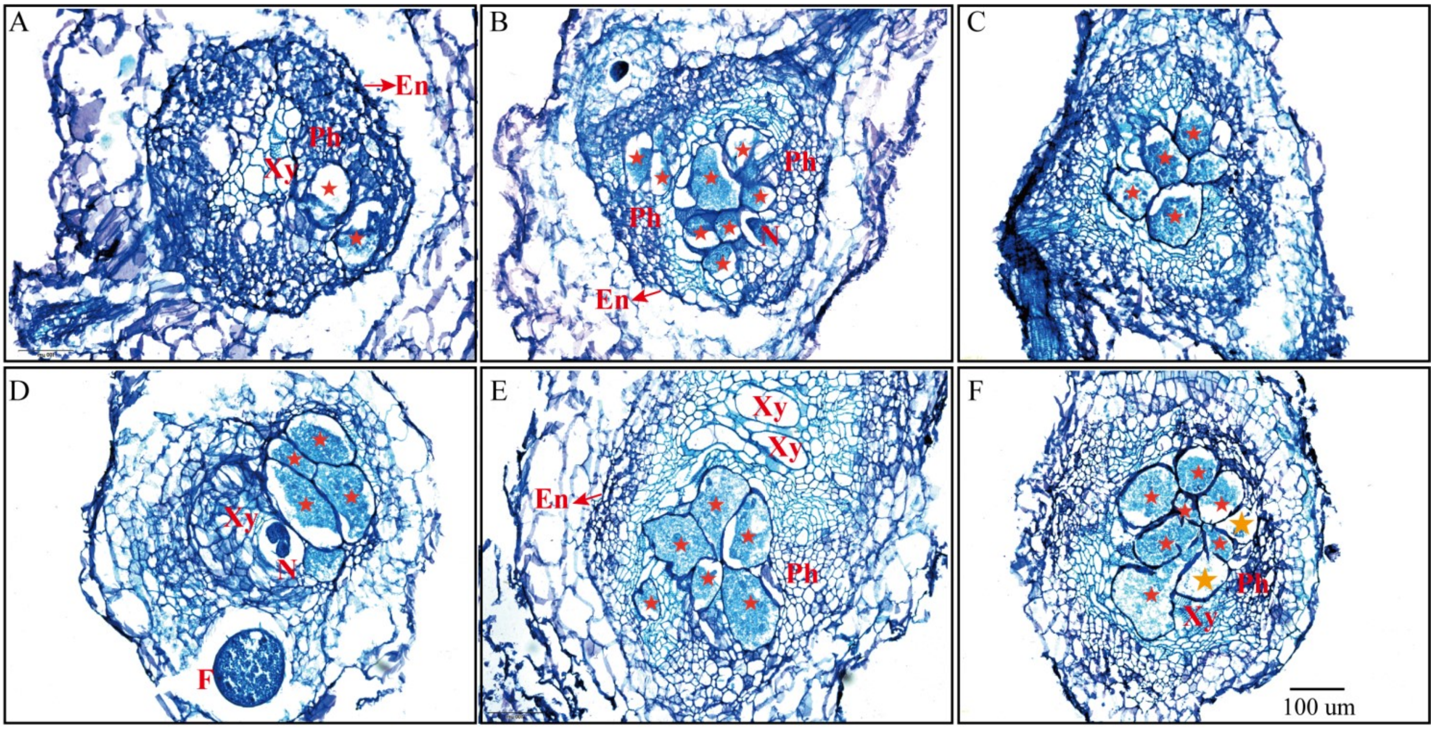

Supplemental figure S2 Cross sections of gall stained with toluidine blue at 7 dpi (A), 14 dpi (B), 21 dpi (C), 28 dpi (D), 35 dpi (E), and 42 dpi (F).

Red pentagram indicates giant cell; Yellow pentagram indicates giant cell with vage. En, endodermis; Ph, phloem; Xy, xylem; N, nematode; F, female nematode.

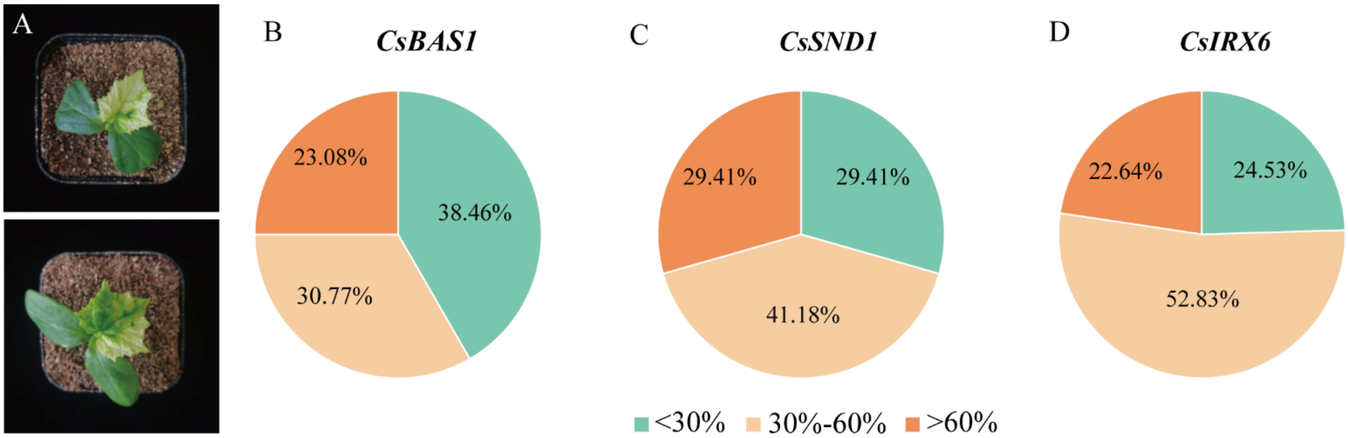

Supplemental figure S3 *TRSV*-mediated VIGS system is used to identify the functions of *CsBAS1*, *CsSND1*, and *CsIRX6* in cucumber infected by *M. incognita*. A, Phytoene desaturase gene (PDS) silenced plants are used as a positive control of VIGS experiment. B, C, and D, the expression levels distribution of *CsBAS1*, *CsSND1*, and *CsIRX6* in each silenced cucumber roots compare with *TRSV::00* control. <30% indicates thatthe gene expression level in the silenced lines is less than 30% of that in the control, corresponding to a silencing efficiency of over 70%; 30%-60% indicates that the gene expression level in the silenced lines is between 30% and 60% of that in the control; >60% indicates that the gene expression level in the silenced lines is 60% of that in the control.
